# Pulmonary alveolar proteinosis postlung transplantation: A causation conundrum

**DOI:** 10.1016/j.jhlto.2023.100002

**Published:** 2023-08-06

**Authors:** Harold Matos, Ashish Maskey, Suresh Keshavamurthy, Jordan Miller, Sravanthi Nandavaram

**Affiliations:** a Department of Medicine, University of Kentucky, Lexington, Kentucky; b Department of Surgery, University of Kentucky, Lexington, Kentucky

**Keywords:** pulmonary, alveolar, proteinosis, lung, transplant, surfactant

## Abstract

Pulmonary alveolar proteinosis (PAP) is a rare pulmonary disorder caused by defective granulocyte–macrophage colony-stimulating factor signaling, leading to abnormalities of macrophage metabolic and immune functions, with resultant impaired surfactant metabolism and its accumulation within the alveoli. PAP can relapse in patients who underwent lung transplantation for PAP related to genetic defects. However, its occurrence is exceedingly rare in lung allografts of patients who underwent lung transplantation for other primary end-stage lung disease. Prompt diagnosis and appropriate treatment strategy are crucial to prevent the decline in the allograft function and to avoid unnecessary empiric treatments for rejection and/ infection. Here we present a case of PAP in a patient, 8 months postbilateral lung transplantation for COVID-19 fibrosis.

Pulmonary alveolar proteinosis (PAP) is a rare and unusual entity characterized by impaired surfactant metabolism and its accumulation within the alveoli. It is caused by defective granulocyte–macrophage colony-stimulating factor (GM-CSF) signaling, leading to abnormalities of macrophage metabolic and immune functions, which include surfactant homeostasis within alveoli.^1^ Primary and secondary causes have been identified, with autoimmune mechanisms being the most common. PAP is known to recur in patients who undergo lung transplantation for primary PAP.^1^ However, to this date, and to our knowledge, only a small number of new-onset cases of PAP following lung transplantation have been reported.

We present a case of PAP which was a diagnostic conundrum and propose a mechanism which led to its occurrence in our patient.

## Case presentation

A 37-year-old male underwent bilateral lung transplantation for severe COVID-19 disease and fibrosis. The induction immunosuppression regimen consisted of basiliximab, mycophenolate, and methylprednisolone and was subsequently maintained on tacrolimus, mycophenolic acid, and prednisone. Following transplantation, the clinical course was uneventful, with good graft function. Surveillance bronchoscopies and transbronchial biopsies did not reveal any signs of rejection or airway complications. Bronchoalveolar lavage from the first surveillance bronchoscopy showed *Mycobacterium fortuitum* for which the patient was treated with azithromycin, inhaled amikacin, linezolid, and moxifloxacin. Eight months post-transplant, patient presented with complaints of cough and shortness of breath, associated with a decline in his forced expiratory volume in 1 second by 19% and forced vital capacity by 16%. Chest imaging revealed upper lobe predominant diffuse bilateral ground glass opacities. Bronchoscopy revealed clear airways, without any evidence of lesions or mucosal damage. Bronchoalveolar lavage fluid was cloudy, but not milky. Transbronchial biopsies revealed focal hyaline membrane formation consistent with acute alveolar damage. There was no evidence of acute cellular rejection. Gomori methenamine silver did not reveal the presence of fungal elements or *Pneumocystis jiroveci*. Gram and Fite stains for bacteria and acid-fast organisms, as well as immunostains for cytomegalovirus, adenovirus, and herpes simplex virus were all negative. An extensive workup, including testing for donor-specific antibodies, infectious and inflammatory causes, and dysphagia were all negative. Follow-up mycobacterial stain and cultures of sputum remained negative. Treatment for *M. fortuitum* was discontinued. Due to the persistence of radiographic and clinical abnormalities, with an otherwise nonrevealing workup, right video-assisted thoracoscopic wedge resection of the superior segment of the right lower lobe was performed. Wedge biopsies revealed alveolar spaces containing variable amounts of purplish-gray, fibrinous-like material associated with intra-alveolar foamy-appearing macrophages and aggregates of eosinophilic material. These aggregates strongly stained positive for PAS with and without amylase digestion, consistent with a diagnosis of PAP. Surfactant protein D levels were normal at 77 ng/mL. Anti-GM-CSF antibody testing was negative. The patient’s symptoms, clinical and radiographic findings worsened, and the decision was made to stop azithromycin and switch the calcineurin inhibitor from tacrolimus to cyclosporine. Whole lung lavage could not be performed given the patient’s clinical condition. Over the next 3–4 months, the patient’s symptoms and hypoxia improved, the ground glass opacities on the imaging resolved, and the graft function improved back to his previous baseline.

## Discussion

PAP is an exceedingly rare complication among the conditions that may adversely impact the graft function in lung transplant recipients. In general, it can develop at any time within the first-year post-transplantation, and fatality risk directly correlates with earlier onset.^2^ Due to a broad list of differentials, including infection and rejection, lack of bronchoalveolar lavage fluid routine stain assessment for PAP and the heterogenous radiologic manifestations of PAP can result in a delayed and a challenging diagnosis.^2^

Only a small number of de novo PAP cases have been reported among lung transplant recipients since the 1990s.^3^ Interestingly, a lack of involvement of the native lung has been observed in some recipients of single-lung transplantation, suggesting the presence of alveolar macrophage injury linked to a pathogenic process specific to graft.^2^ Relevant to this, is the finding that donor alveolar macrophages can persist for several years.^4^ Impaired lymphatic drainage is postulated as another contributor.^5^ Immunosuppression has also been assigned a pathogenic role in cases of symptomatic PAP among solid-organ transplant recipients,^2^, ^5^, ^6^ most commonly in those receiving mammalian target of rapamycin (mTOR) inhibitors.^2^, ^7^ The actual mechanism by which PAP may occur de novo with the use of these drugs is not clear.^8^

In our case, the diagnosis and identification of the causative agent was challenging due to the indistinct clinical and radiographic findings. Our patient never received mTOR inhibitors, infectious workup was nonrevealing, and autoantibodies to GM-CSF were negative. We did not perform the whole lung lavage because of hypoxia. In retrospect, a notable phenomenon was slow but distinct pattern of resolution following first the withdrawal of azithromycin and subsequent replacement of tacrolimus with cyclosporine. With this in mind, we hypothesize that the synergistic inhibition of GM-CSF by both macrolide antibiotics, tacrolimus and azithromycin, may have resulted in the development of PAP in our patient.

Tacrolimus-mediated calcineurin inhibition reduces transcriptional activation of GM-CSF.^9^ It is also chemically related to rapamycin, and both can selectively promote the degradation of GM-CSF messenger RNA.^9^, ^10^ Azithromycin, which has well-known immunomodulatory effects, has been specifically shown to alter macrophage function leading to decreased GM-CSF levels and function.^11^, ^12^ Theoretically in combination, drugs that reduce GM-CSF signaling in transplanted lungs could therefore impair macrophage function and, consequentially, affect surfactant clearance. Divithotawela et al reported the development of PAP secondary to immunosuppression-induced macrophage dysfunction. Adjustment of the immunosuppressive regime to the lowest acceptable level was followed by PAP resolution.^13^

**Figure 1:**
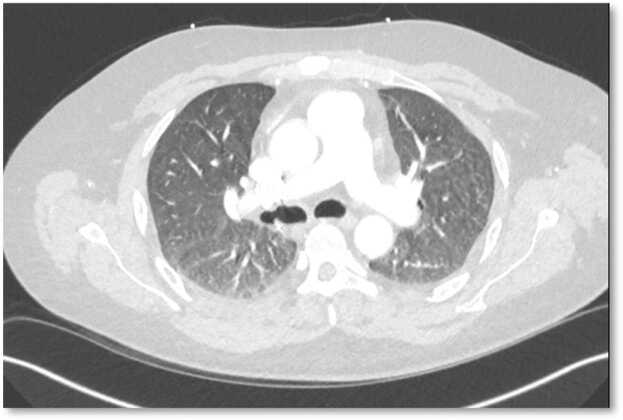
Chest CT axial image with demonstration of the diffuse bilateral ground glass opacities.

**Figure 2:**
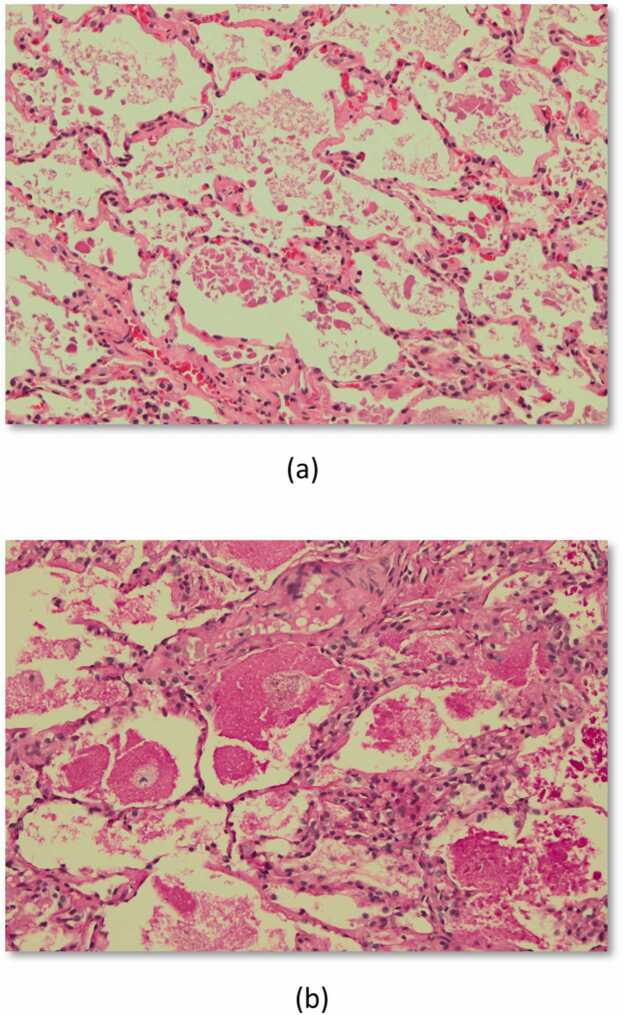
(a) H&E-stained section of right lower lobe superior segment wedge biopsy alveolar parenchyma demonstrating the presence of purplish gray eosinophilic fibrinous-like material and intra-alveolar foamy macrophages. (b) Periodic Acid Schiff-stained section of right lower lobe superior segment wedge biopsy alveolar parenchyma.

**Figure 3:**
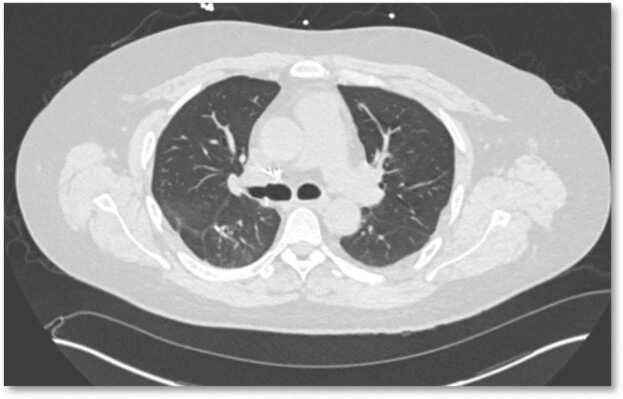
Follow-up chest CT axial image with demonstration of resolution of the bilateral ground glass opacities.

In summary, we highlight a case of de novo secondary PAP in a lung transplant recipient, in the absence of better-established causes, raising a plausible correlation with the exposure to immunomodulatory drugs of the macrolide class, which possess inherent macrophage-modifying effects. Patient’s symptoms, hypoxia, and radiographic findings resolved, and allograft function improved back to patient’s previous baseline with cessation of azithromycin and by changing the immunosuppression, without the need for whole lung lavage. Further research is warranted to support and better define this mechanism (Figure 1, Figure 2, Figure 3).
