# Corrigendum to ‘Pulmonary alveolar proteinosis postlung transplantation: A causation conundrum’ [JHLT Open 1 (2023) 100002]

**DOI:** 10.1016/j.jhlto.2024.100119

**Published:** 2024-07-08

**Authors:** Harold Matos, Ashish Maskey, Suresh Keshavamurthy, Jordan Miller, Sravanthi Nandavaram

**Affiliations:** a Department of Medicine, University of Kentucky, Lexington, Kentucky; b Department of Surgery, University of Kentucky, Lexington, Kentucky

The authors regret that the patient consent statement was not published in the article.

## Patient consent

The authors attest that they obtained written informed consent for patient information to be published in this article.

The authors would like to apologize for any inconvenience caused.

